# Investigating the Nexus of NLRP3 Inflammasomes and COVID-19 Pathogenesis: Unraveling Molecular Triggers and Therapeutic Strategies

**DOI:** 10.3390/v16020213

**Published:** 2024-01-31

**Authors:** Qun He, Da Hu, Fuqiang Zheng, Wenxuan Chen, Kanghong Hu, Jinbiao Liu, Chenguang Yao, Hanluo Li, Yanhong Wei

**Affiliations:** 1 Sino-German Biomedical Center, National “111” Center for Cellular Regulation and Molecular Pharmaceutics, Key Laboratory of Fermentation Engineering (Ministry of Education), Hubei University of Technology, Wuhan 430068, China; 18396954409@163.com (Q.H.); zfq15637689953@163.com (F.Z.); 18295118694@163.com (W.C.); hukh@hbut.edu.cn (K.H.); jinbiaoliu@hbut.edu.cn (J.L.); yaochenguang@hbut.edu.cn (C.Y.); lihanluo@hbut.edu.cn (H.L.); 2 Sinopharm Animal Health Corporation Ltd., Wuhan 430075, China; hdaassdd@163.com

**Keywords:** NLRP3 inflammasome, SARS-CoV-2, COVID-19, cytokine storm, therapeutic targets

## Abstract

The coronavirus disease 2019 (COVID-19) global pandemic, caused by severe acute respiratory syndrome coronavirus type 2 (SARS-CoV-2), has been marked by severe cases demonstrating a “cytokine storm”, an upsurge of pro-inflammatory cytokines in the bloodstream. NLRP3 inflammasomes, integral to the innate immune system, are speculated to be activated by SARS-CoV-2 within host cells. This review investigates the potential correlation between NLRP3 inflammasomes and COVID-19, exploring the cellular and molecular mechanisms through which SARS-CoV-2 triggers their activation. Furthermore, promising strategies targeting NLRP3 inflammasomes are proposed to mitigate the excessive inflammatory response provoked by SARS-CoV-2 infection. By synthesizing existing studies, this paper offers insights into NLRP3 as a therapeutic target, elucidating the interplay between COVID-19 and its pathophysiology. It serves as a valuable reference for future clinical approaches in addressing COVID-19 by targeting NLRP3, thus providing potential avenues for therapeutic intervention.

## 1. Introduction

Coronavirus disease 2019 (COVID-19), stemming from severe acute respiratory syndrome coronavirus 2 (SARS-CoV-2), is classified within the Betacoronavirus genus of the Coronaviridae family. This enveloped virus bears a single-stranded, positive-sense RNA genome of approximately 30 kilobases in length [1]. Its genetic makeup comprises two-thirds of the genome devoted to encoding substantial polyprotein precursors, open reading frame (ORF) 1 and ORF1b, which subsequently undergo proteolytic cleavage to yield 16 non-structural proteins [2]. The remaining one-third of the genome encodes key structural proteins—spike (S), envelope (E), matrix (M), and nucleocapsid (N) proteins—alongside non-structural proteins and a series of accessory proteins (3a, 3b, 6, 7a, 7b, 8a, 8b, and 9b) [3]. The SARS-CoV-2 pandemic, characterized by its elevated mortality rates, has emerged as a pressing global health concern. This viral infection inflicts damage on multiple organs, including the lungs, heart, blood vessels, kidneys, and intestines, and can precipitate a critical complication known as a “cytokine storm”. This phenomenon triggers an uncontrolled hyperactivation of the innate immune response, resulting in severe implications. The global spread of the COVID-19 pandemic has sparked widespread apprehension regarding viral infection and its underlying pathophysiological mechanisms. Inflammation stands as a pivotal factor contributing to the escalation of severe complications in patients afflicted with the COVID-19 [4].

Inflammasomes represent a critical facet of the innate immune system, amplifying inflammatory responses by upregulating the production of interleukin-1 beta (IL-1β), IL-18, and gasdermin. Pattern-recognition receptors (PRRs), including NACHT, LRR, and PYD domain-containing protein 1 (NLRP1), NLRP3, NLR family CARD domain-containing protein 4 (NLRC4), and absent in melanoma 2 (AIM2), facilitate the recruitment of the apoptosis-associated speck-like protein (ASC) and caspase-1 to assemble inflammasomes [5]. Additionally, NLRP6, RIG-I, AIM2, PYRIN, and NLRC5 contribute to the assembly of these inflammatory complexes. Indeed, inflammasomes are pivotal in the pathogenesis of numerous infectious diseases characterized by detrimental inflammation. Extensive studies underscore the hyperactivation of inflammasomes during viral infections, culminating in systemic and damaging inflammatory responses in patients. Among the various types of inflammasomes identified, NLRP3 inflammasomes have garnered significant attention and are pivotal in both inflammation and antiviral responses [6]. The NLRP3 inflammasome, an intracellular protein complex, stands as a vital component in the orchestration of inflammatory regulation and immune responses. The NLRP3 protein, a constituent of the nod-like receptor protein (NLR) family, operates in concert with the ASC protein (Apoptosis-associated speck-like protein containing a CARD) and pro-inflammatory precursor cytokines, notably IL-1β and IL-18. These entities engage in intricate cellular interactions, coalescing to form a complex assembly. Primarily, their function revolves around detecting danger signals and modulating inflammatory responses. Upon cellular stimulation by various triggers such as injury, infection, or oxidative stress, the NLRP3 protein undergoes conformational alterations. This leads to its activation and subsequent assembly, culminating in the formation of inflammasomes. This intricate process encompasses the regulation of diverse signaling pathways, including calcium dynamics, mitochondrial impairment, oxidative stress, and the accumulation of intracellular metabolites. The activation of the NLRP3 inflammasome instigates a cascade of biological reactions. Foremost among these is the processing and release of pro-inflammatory cytokines—IL-1β and IL-18. These cytokines wield pivotal influence within the immune system by orchestrating inflammatory responses, bolstering immune cell activation and proliferation, and contributing to the regulation of physiological processes such as apoptosis.

The involvement of the NLRP3 inflammasome in SARS-CoV-2 infection and the identification of potential inhibitors have become central themes in contemporary medical research. Investigations have indicated a plausible link between the dysregulated immune responses triggered by the novel coronavirus and the potential activation of the NLRP3 inflammasome. Hence, comprehending the pathological role of NLRP3 in SARS-CoV-2 infection, elucidating its influence on the inflammatory process, and assessing the current status of NLRP3 inhibitors have gained paramount importance. This article aims to delve into the potential mechanisms underlying the involvement of NLRP3 inflammasomes in novel coronavirus infection, shedding light on how they modulate the inflammatory cascade. Additionally, it will scrutinize the ongoing research advancements concerning NLRP3 inhibitors for COVID-19 treatment, aiming to offer novel insights and potential therapeutic strategies for managing this disease. Amid the global response to the challenges posed by COVID-19, a comprehensive understanding of the NLRP3 inflammasome’s mechanisms and the evolving landscape of related inhibitors holds significant promise in guiding treatment approaches and curbing disease progression.

## 2. Activation of NLRP3

The physiological significance of the NLRP3 inflammasome lies in its pivotal role in regulating the inflammatory response. Upon activation, NLRP3 interacts with the PYD domain of apoptosis-associated ASC [7], subsequently binding with caspase-1 to form inflammasomes. This assembly triggers the cleavage and activation of caspase-1, fostering pyroptosis, and prompting the secretion of pro-inflammatory factors such as IL-1β or IL-8 [8]. These cytokines play a crucial role in the immune response, aiding the host in combating infections. However, excessive activation can contribute to the onset of inflammatory diseases like rheumatoid arthritis, inflammatory bowel disease, among others. Beyond its involvement in immune responses, NLRP3 inflammasomes are intricately linked to the initiation and progression of various diseases, including metabolic disorders like type 2 diabetes [9], atherosclerosis [10], and neurodegenerative conditions [11]. Consequently, studying NLRP3 inflammasomes not only facilitates an understanding of immune-regulation mechanisms but also unveils a promising new target for treating associated diseases.

**Figure 1:**
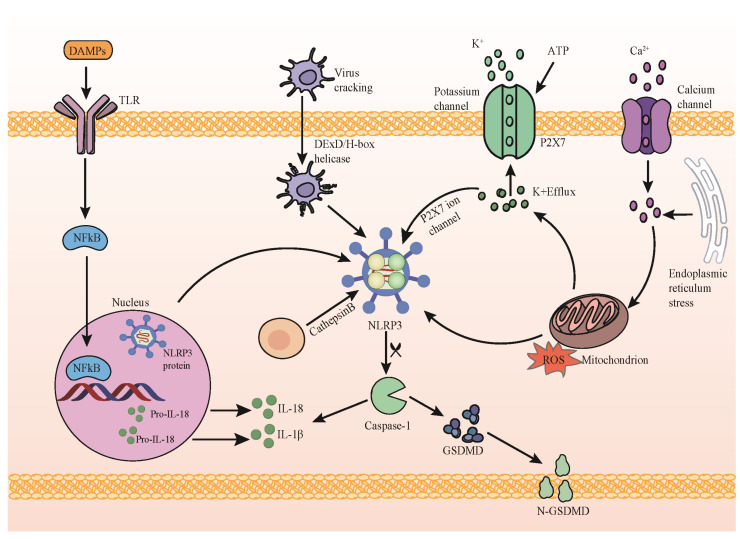
NLRP3 inflammasome activation pathway. Initiation: stimulation of the membrane receptor TLR by damage-associated molecular patterns (DAMPs) initiates NF-κB translocation, inducing the upregulation of components associated with the NLRP3 inflammasome. Activation: key cellular signals triggered by activated NLRP3 include potassium (K^+^) efflux, calcium (Ca^2+^) efflux, reactive oxygen species (ROS) production, mitochondrial impairment, and lysosome destabilization. Activated NLRP3 catalyzes procaspase-1, leading to the cleavage of procaspase-1 into active caspase-1. Caspase-1 then cleaves pro-IL-1β and pro-IL-18 into their active forms. Additionally, GSDMD cleavage by caspase-1 facilitates the release of IL-1β and IL-18.

NLRP3 activation stands as a fundamental process in immune system inflammatory regulation, intricately involving complex cellular signal pathways and protein interactions [12]. While most inflammatory complexes respond to highly specific agonists, NLRP3 activation demonstrates a unique sensitivity to diverse stimuli, including alterations in K^+^ or Cl^−^ concentrations, calcium fluctuations, lysosomal disruption, mitochondrial dysfunction, metabolic shifts, and trans-Golgi disintegration (Figure 1). The activation of NLRP3 unfolds in two principal stages [13]. Initially, cells perceive an array of pathological stimuli, encompassing bacterial infections, oxidative stress, and intracellular metabolite changes. These triggers prompt intracellular K^+^ ion efflux, induce mitochondrial impairment, and release signaling molecules such as reactive oxygen species (ROS). These alterations significantly impact the conformation or aggregation state of NLRP3, culminating in its activation. Activated NLRP3 subsequently interfaces with the intracellular adapter protein ASC, culminating in the formation of polymerized inflammatory bodies. This step marks a pivotal juncture in NLRP3 activation, establishing a platform for subsequent events. The subsequent stage involves a cascade of enzymatic reactions within the inflammatory bodies. Within these structures, ASC polymerization provides a scaffold that triggers the activation of the cascade enzymes. This activation event initiates a cascade of enzymatic reactions that are integral to the inflammatory response. This understanding of NLRP3 activation dynamics sheds light on its unique responsiveness to diverse stimuli, highlighting its role as a crucial mediator of inflammatory regulation within the immune system. Upon activation, Cascade enzymes facilitate the cleavage of pro-inflammatory cytokine precursors, such as IL-1β and IL-18, converting them into their active forms. Subsequently, these active cytokines are released extracellularly, engaging in inflammatory regulation and immune responses [14]. It is noteworthy that the specifics of NLRP3 activation may vary depending on cell types, external stimuli, and the pathological context. The excessive or aberrant activation of NLRP3 is strongly associated with the onset of various inflammatory diseases, autoimmune conditions, and metabolic disorders.

The activation of the NLRP3 inflammasome plays a dual role in the process of virus infection. On the one hand, the regulation of inflammatory responses by NLRP3 activation can affect the function of immune cells, thus affecting the antivirus. NLRP3 plays a potential role in a variety of viral infections. Influenza virus infection is a significant example; the activation of the NLRP3 inflammasome is closely related to the immune response. In the early stages of IAV infection (the first five days after infection), the activation of NLRP3 triggers the release of inflammatory factors such as IL-1 β and IL-18, which help recruit immune cells and improve their ability to fight the virus [15]. Cytomegalovirus infection also shows signs that NLRP3 may play an antiviral effect. NLRP3 activation is accompanied by the release of pro-inflammatory cytokines, which helps to improve the virus clearance ability of immune cells [16]. This suggests that NLRP3 may play a similar regulatory role in different types of viral infections. In addition, adenovirus infection shows that the activation of NLRP3 is related to the regulation of an inflammatory response. NLRP3 activation affects the immune response by regulating the release of inflammatory mediators and plays an antiviral role in adenovirus infection [17]. These examples suggest that NLRP3 inflammasomes regulate the inflammatory response and affect immune cell function in different viral infections and may have potential antivirus effects. However, more in-depth research is needed to fully understand the specific mechanism of the NLRP3 inflammasome, and its role and impact in different virus infections. On the other hand, excessive NLRP3 inflammasome activation can trigger a cytokine storm, an overdrive of the body’s immune response leading to the release of numerous pro-inflammatory cytokines. This cascade induces tissue inflammation, cellular damage, and even organ failure, contributing significantly to severe infections, autoimmune conditions (e.g., rheumatoid arthritis, systemic lupus erythematosus), and certain inflammatory diseases. Efforts to regulate NLRP3 inflammasome activation serve as a potential strategy to mitigate the intense inflammatory response associated with cytokine storms. Studies explore this avenue as a means to attenuate severe inflammatory conditions. For example, in hepatitis C virus (HCV) infection, NLRP3 activation drives a network of pro-inflammatory cytokines, chemokines, and immunomodulatory genes, exacerbating chronic intrahepatic inflammation and liver injury [18]. Moreover, cytokine storm pathogenesis is implicated in diseases like severe dengue (DENV) [19], where DENV-mediated NLRP3 inflammasome activation prompts IL-1β and IL-18 secretion [20], exacerbating vascular permeability in severe cases [21]. Furthermore, the direct interaction of the SARS-CoV-2 N protein with NLRP3 triggers inflammasome formation, leading to excessive inflammation, acute lung injury, and hastened sepsis-induced mortality [22]. Additionally, a polypeptide analogue of the Hendra virus C protein (HEVC) has been identified to aggregate and activate NLRP3 inflammasomes, inducing severe inflammatory lung and nervous system diseases upon phagocyte uptake [23]. Therefore, a comprehensive exploration into the mechanisms governing NLRP3 activation holds promise in uncovering novel targets and strategies for treating and preventing inflammation-related diseases.

## 3. Functions of NLRP3 Inflammasome in SARS-CoV-2 Infection

### 3.1. Findings in the Context of SARS-CoV-2 Infection

SARS-CoV-2 is a coronavirus that causes COVID-19. The release of a high amount of cytokines leads to severe inflammation and acute damage to multiple organs following SARS-CoV-2 infection [24]. The interaction between the infection mechanism and immune response is very important for disease progression, profoundly influencing disease progression. Emerging studies highlight that SARS-CoV-2 infection prompts the potential activation of NLRP3 inflammasomes within host cells. Notably, an overactive response involving NLRP3 inflammasome activation and the subsequent uncontrolled production of neutrophil extracellular traps (NETs) are regarded as critical facets of severe disease manifestation [25,26,27]. The activation and prolapse of NLRP3 inflammatory bodies were detected in monocytes and macrophages during SARS-CoV-2 infection [28]. In addition, danger signals, such as calcitonin, which have been recently associated with the severity of COVID-19, are known to activate or release NLRP3 inflammasomes as a result of their activation, and may cause prolapse [29].

### 3.2. The Activation of NLRP3 by SARS-CoV-2 Proteins

Previous studies found a positive correlation between increased serum levels of IL-1β, IL-18, and LDH in COVID-19 patients and the severity of the disease, suggesting the involvement of inflammasomes in the pathogenesis of SARS-CoV-2 [30]. Notably, the structural proteins of SARS-CoV-2—spike protein (S) [31], envelope protein (E) [32], and nucleocapsid protein (N) [22]—are closely associated with the activation of NLRP3 inflammasomes. SARS-CoV-2’s nucleocapsid protein (SARS-CoV-2N) has been identified to activate the SREBP cleavage-activating protein (SCAP), inducing its dissociation from the endoplasmic reticulum. This event leads to the activation of SREBP, increased expression of lipogenic genes, and subsequent activation of NLRP3 inflammasomes. Additionally, Trimethylamine N-oxide (TMAO) influences vascular smooth muscle cells (VSMCs) by promoting the translocation of the SCAP–SREBP complex from the endoplasmic reticulum to the Golgi matrix, subsequently fostering NLRP3 inflammasome activation [33]. There is also evidence suggesting the direct binding of SARS-CoV-2N to the NLRP3 protein; the SARS-CoV-2N protein facilitates the interaction between NLRP3 and ASC, consequently promoting the oligomerization of ASC. This interaction gives rise to the formation of a complex, denoted as N-NLRP3-ASC, which triggers the activation of NLRP3 inflammasomes [22], solidifying NLRP3’s pivotal role in coronaviruses. SARS-CoV-2 enters human cells by binding to the angiotensin-converting enzyme 2 (ACE2) receptor, employing its S protein for attachment and entry into the host cells [34,35]. The viral S protein requires priming by transmembrane protease 2 (TMPRSS2) to facilitate its interaction with ACE2, leading to the fusion of viral and cellular membranes. ACE2 [36], found on various cell surfaces, physiologically converts angiotensin II (Ang II) to angiotensin (1–7) (Ang [1–7]) [37]. Notably, SARS-CoV-2 viral proteins, particularly the S protein, bind to ACE2, contributing to the activation of NLRP3 inflammasomes [31]. Several investigations propose that the E protein exhibits a dichotomous influence on the activation of the NLRP3 inflammasome [38]. The SARS-CoV-2 E protein exhibits a dual functionality in orchestrating the activation of inflammasomes. Primarily, it impedes the initiation of inflammasomes in both bone marrow-derived macrophages (BMDM) and human pluripotent stem cell-derived macrophages, thereby diminishing the early-stage activation of NLRP3 inflammasomes during viral infection. Conversely, in the later phases of infection, when inflammasomes are triggered by endotoxins, DAMPs, or other viral factors, the E protein engages with viral RNA, potentially fostering the activation of NLRP3 inflammasomes. Consequently, in more intricate or advanced disease scenarios, the E protein may mediate a biphasic impact throughout viral infection, characterized by initial immunosuppression followed by the subsequent activation of NLRP3 inflammasomes [39]. This nuanced modulation underscores the multifaceted role played by the SARS-CoV-2 E protein in the host’s immune response, offering new insights into the complex interplay between viral components and host inflammatory pathways. The non-structural proteins (NSPs) of SARS-CoV-2 also play a role in affecting NLRP3 activation. NSP1 and NSP13 have been identified to inhibit caspase-1-mediated IL-1β activation. Moreover, the transmembrane pore-forming protein 3a (SARS-CoV3a), a helper protein, is positioned on the plasma membrane and its ion channel activity is pivotal for NLRP3 inflammasome activation. This protein mediates the secretion of IL-1β, requiring K^+^ outflow and mitochondrial ROS production [40,41], elucidating the potential of the NLRP3 inflammasome as a molecular target for COVID-19 treatment. Furthermore, the genomic architectures of SARS-CoV (severe acute respiratory syndrome coronavirus) and SARS-CoV-2 (novel coronavirus) exhibit substantial similarities. Both genomes comprise multiple open reading frames (ORFs) responsible for encoding a spectrum of structural and non-structural proteins. The identity and similarity of ORF8 in the SARS-CoV-2 and ORF8a in SARS-CoV are 38.9 and 77.8, respectively. The ORF8 protein of SARS-CoV-2 is 44.4% identical and 66.7% similar to ORF8b of SARS-CoV [42]. The SARS-CoV open reading frame 8b (ORF8b) activates the intracellular stress pathway by forming insoluble aggregates. ORF8b directly interacts with the leucine-rich repeat domain of NLRP3, co-localizing with NLRP3 and ASC in cytoplasmic structures, thereby directly activating NLRP3 inflammasomes, targeting innate immunity [43]. The activation of inflammasomes exerts two major effects: it activates caspase-1-mediated processing and the secretion of pro-inflammatory cytokines IL-1β and IL-18, and induces inflammatory cell death and pyroptosis via a protein called gasdermin D [44]. Pyroptosis, a distinctive mode of inflammatory programmed cell death, is characterized by the rupture of the cell membrane. The manifestation of pyroptotic cell demise serves a crucial role in clearing infected cells and releasing cellular contents, including pro-inflammatory factors, thereby intensifying the immune response. These intricate molecular processes are integral in the host’s defense against infections and the preservation of immune homeostasis. However, the hyperactivation of inflammasomes is intricately linked to the onset and progression of specific diseases. Consequently, the investigation into the regulatory mechanisms governing inflammasomes has emerged as a promising focus for potential therapeutic strategies in the context of inflammatory diseases.

### 3.3. Role of NLRP3 Inflammasome in COVID-19 Immune Activation

In the host’s innate immune response to coronavirus infection, a pivotal initial step, involves the production of type I and III interferons (interferon-I and interferon-III, respectively), along with pro-inflammatory cytokines and chemokines [45]. Following the recognition of viral pathogen-associated molecular patterns (PAMPs) and/or host damage-associated molecular patterns (DAMPs), various cell types employ specialized pattern-recognition receptors (PRRs) to generate interferon-I and interferon-III [45]. For SARS-CoV-2, RNA-based replication intermediates serve as the primary viral PAMPs, detected by RIG-I-like receptors and Toll-like receptors (RLRs and TLRs), respectively. The engagement of TLRs with the appropriate viral ligands initiates the recruitment of MyD88 or TRIF cell adapter proteins, triggering NF-κB and IRF activation through a cascading signaling pathway [46]. The activation of these transcription factors, coupled with factors related to cellular stress, culminates in the assembly of a large complex known as an enhancer. This enhancer binds upstream of the transcriptional initiation sites for interferon I members, particularly interferon β, and interferon III members, such as interferon lambda-1, interferon lambda-2, and interferon lambda-3, instigating a robust antiviral response [47]. An intricate interplay exists between interferons and NLRP3 inflammasomes. Beyond interferons, the sensing of viral RNA by PRRs induces the production of various cytokines and chemokines, activating signal cascades, notably the NF-κB pathway. This cascade ultimately leads to the expression of diverse cytokines and chemokines, triggering the activation of NLRP3 inflammasomes. This activation facilitates the cleavage and activation of cytokines like IL-1β and IL-18 [30].

The progression of COVID-19 involves intricate immune responses, notably the activation of NLRP3 inflammasomes. Upon SARS-CoV-2 infection, host cells undergo a stress response, potentially altering the intracellular milieu or releasing virus-associated molecules. These aberrant signals prompt NLRP3 inflammasome activation, wherein the NLRP3 protein interacts with ASC (adaptor protein) and precursor caspase-1, culminating in active inflammasomes. Subsequently, activated inflammasomes trigger precursor caspase-1 activation, facilitating the cleavage and maturation of pro-inflammatory cytokines like IL-1β and IL-18. The release of IL-1β and IL-18 swiftly initiates immune responses, eliciting chemical signals at the site of infection and recruiting macrophages, neutrophils, and other inflammatory cells. Consequently, these cells release additional inflammatory mediators (e.g., TNF-α, IL-6), intensifying the inflammatory cascade. This cascade not only incites cellular damage and tissue inflammation but also prompts immune system regulatory mechanisms involving specialized cells such as T cells and B cells. While NLRP3 inflammasome activation serves as a catalyst for immune responses, its excessive activation may precipitate an exaggerated inflammatory response in COVID-19, potentially contributing to severe illness and complications.

### 3.4. Effect of NLRP3 on Cytokine Storms in SARS-CoV-2 Infection

The “cytokine storm” is the main cause of COVID-19‘s poor clinical outcomes. The cytokine storm caused by novel coronavirus infection involves a variety of complex biological mechanisms, which promote the accumulation and activation of immune cells, causing them to move quickly to the site of infection. Excessive cell activation and infiltration may incite an inflammatory cascade in the lungs and other tissues, exacerbating disease severity. Moreover, the abrupt release of numerous inflammatory mediators can surpass normal immune regulation, leading to uncontrolled inflammation and consequential cell and tissue damage, notably in the lungs, possibly culminating in acute respiratory distress syndrome (ARDS). Cytokine storms can disrupt immune system balance, either overactivating or inhibiting it, potentially fostering autoimmunity and rendering the body more susceptible to secondary infections. The cytokine storm in patients with COVID-19 is implicated in deleterious effects on vascular endothelial cells, mediated through diverse mechanisms encompassing endothelial cell activation, inflammation, aberrant blood coagulation, and oxidative stress. Overall, the NLRP3 inflammasome’s activation, contributing to the cytokine storm in novel coronavirus infection, triggers an unbridled immune response, tissue damage, and systemic complications via the release of inflammatory mediators, immune dysregulation, and vascular endothelial inflammation and dysfunction, profoundly affecting the infected individuals’ health.

### 3.5. NLRP3’s Role in SARS-CoV-2-Induced Multiorgan Dysfunctions

**Figure 2:**
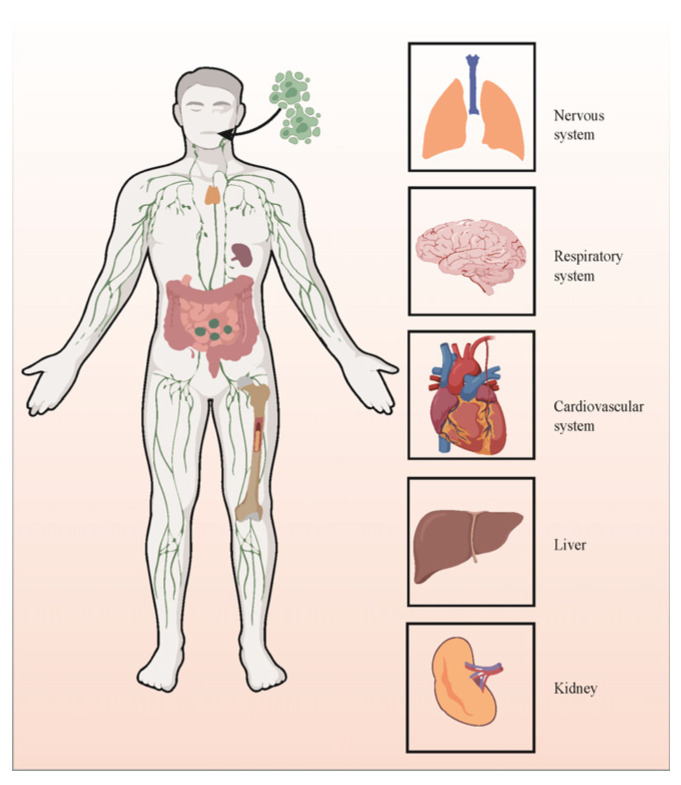
NLRP3’s role in SARS-CoV-2-induced multiorgan dysfunctions.

Furthermore, the involvement of the NLRP3 inflammasome is paramount in the pathogenesis of multi-organ damage induced by COVID-19. Patients infected with SARS-CoV-2 exhibit damage across various organs, including the lungs, brain, liver, kidney, and spleen (Figure 2). Severe cases of COVID-19 are associated with the progression of intense fibrotic responses, elevating the risk of idiopathic pulmonary fibrosis. Notably, NLRP3 inflammasomes are directly implicated in the onset of pulmonary fibrosis. Autopsy findings and animal models have consistently affirmed that the aberrant expression of NLRP3 inflammasomes significantly contributes to the pathophysiological mechanisms underlying acute respiratory distress syndrome (ARDS). Pathways associated with NLRP3 result in severe clinical manifestations, tissue necrosis, heightened moisture, and intense pulmonary inflammation [48]. The neurological manifestations of SARS-CoV-2 are well-documented, encompassing a spectrum of neuropsychological disorders. Central nervous tissue involvement in COVID-19 primarily engages astrocytes and microglia, releasing pro-inflammatory cytokines and inciting neuroinflammation [49]. The pathogenicity of SARS-CoV-2 in the brain correlates with the activation of microglial NLRP3 inflammasomes. The activation of caspase-1 mediated by NLRP3 inflammasomes escalates the cleavage of IL-18 and IL-1β from their precursors, instigating their release. Consequently, activated caspase-1 can disrupt the blood–brain barrier, inciting a neuroinflammatory response [50]. Elevated IL levels stimulate neurons, astrocytes, and microglia to initiate the production of additional pro-inflammatory mediators, thereby instigating neuroinflammation. Notably, liver-related symptoms have been observed in COVID-19 patients, with hepatic sinusoid and pericellular fibrosis detected in autopsy specimens [51]. Under inflammatory conditions, the activation of Kupffer cells, through various mechanisms, can precipitate liver injury and fibrosis via the dysregulation of NLRP3 inflammasomes and excessive IL-1β production [52]. Furthermore, SARS-CoV-2 may infect kidney cells in COVID-19 patients, leading to kidney injury. In cases of SARS-CoV-2 infection, the infiltration of pro-inflammatory cells, such as CD68+ macrophages, into the renal tubulointerstitium has been observed [53]. Macrophage infiltration significantly contributes to inflammation, fibrosis, and renal injury. While there are no specific data supporting the direct involvement of NLRP3 inflammasomes in the pathogenesis of SARS-CoV-2-induced acute renal failure (ARF), certain studies suggest that the aberrant activation of NLRP3 inflammasomes is associated with inflammatory diseases related to ARF [54].

Hence, the modulation of NLRP3 inflammasomes emerges as a potential avenue for treating the inflammatory response induced by SARS-CoV-2 infection. Nevertheless, comprehensive studies and clinical trials are essential to substantiate the precise mechanisms underlying NLRP3 inflammasomes during viral infections. These investigations are critical not only to elucidate their functioning but also to assess the efficacy and safety of therapeutic interventions. Confirming the intricate involvement of NLRP3 in viral infections, particularly SARS-CoV-2, is pivotal for designing targeted therapeutic approaches. Thorough research efforts will not only unveil the underlying mechanisms but also aid in the development of safe and efficient treatments aimed at modulating NLRP3-mediated inflammatory responses triggered by SARS-CoV-2. Such endeavors are indispensable for navigating the complexities of the immune–inflammatory response and advancing potential therapies to mitigate the severity of COVID-19.

### 3.6. The Role of Key Pro-Inflammatory Factors IL-18 and IL-1β Downstream of NLRP3 in SARS-CoV-2 Infection

As the key pro-inflammatory cytokines downstream of NLRP3, interleukin-18 (IL-18) and interleukin-1β (IL-1β) are considered to play an important role in the immune response against SARS-CoV-2 infection. Studies have shown that these cytokines play a dual role in immune regulation in the process of infection. The elevated levels of IL-18 and IL-1β were observed in patients with COVID-19, which were related to the severity of the disease. These cytokines promote the activation of a variety of immune cells, including T cells, macrophages, and natural killer cells, and contribute to the initial antiviral response [55]. However, the excessive or dysfunctional release of IL-18 and IL-1β leads to excessive inflammation, exacerbates tissue damage, and leads to cytokine storms observed in severe COVID-19 cases [56]. Therapeutic interventions targeting IL-18 and IL-1β signaling pathways are being studied to alleviate cytokine-driven pathological processes and are expected to improve the clinical prognosis of patients with COVID-19. IL-18 and IL-1β are the main outputs of activated NLRP3. Together with NLRP3, they participate in the regulation of the inflammatory response and immune regulation. An in-depth understanding of the exact role and regulatory mechanism of IL-18 and IL-1β in SARS-CoV-2 infection is very important for the formulation of targeted treatment strategies and the effective management of COVID-19.

The intricate relationship between the heightened production of interleukin-1 (IL-1) in severe COVID-19 cases and the activation of NLRP3 inflammatory bodies has prompted extensive exploration within the scientific community to devise novel treatment modalities. Currently, IL-1 antagonists, such as Anakinra and canakinumab, are emerging as promising interventions and are undergoing thorough investigation in various clinical trials [57]. Anakinra, in particular, holds significant promise as a treatment option, anticipated to enhance clinical outcomes by controlling monocyte proliferation and inhibiting the activation of NLRP3 inflammatory bodies. Notably, the application of Anakinra to obstruct the IL-1 signal in patients with CMMLKRASmut has successfully mitigated the overactivation of NLRP3 inflammatory bodies, alleviating detrimental inflammatory consequences [58]. Canakinumab, a human monoclonal antibody, demonstrates efficacy in preventing subsequent pro-inflammatory signaling events by disrupting the interaction between IL-1β and IL-1R [57]. The overarching objective of these antagonists is to ameliorate the adverse effects associated with cytokine storms, including the activation of NLRP3 inflammatory bodies. The NLRP3 inflammatory body, serving as a multiprotein complex, assumes a pivotal role in the maturation and release of IL-1β, thereby propelling the inflammatory cascade. The inhibition of IL-1 production disrupts the positive feedback cycle that precipitates excessive inflammation, offering a strategic approach to intervention. NLRP3 inflammatory bodies function as sensors for cellular stress and danger signals, with disruptions in their regulation implicated in various inflammatory diseases, including those linked to severe viral infections like COVID-19. The findings from these studies hold valuable implications for the prospective treatment of severe COVID-19 cases and other inflammatory conditions, presenting a novel avenue for precise intervention and the amelioration of patient conditions.

## 4. Compounds Targeting NLRP3 in the Treatment of SARS-CoV-2 Infection

Currently, ongoing studies are investigating the potential use of NLRP3 inhibitors to modulate the cytokine storms triggered by novel coronavirus infection, aiming to mitigate inflammation and associated pathophysiological processes. We present a compilation of pharmacological agents targeting NLRP3 inflammasomes for COVID-19 treatment (Table 1) and delineate their respective mechanisms of action.

### 4.1. Synthetic Compounds

#### 4.1.1. Alkenyl Sulfonylurea Derivative 7

The alkenyl sulfonylurea derivative 7 has demonstrated high oral bioavailability as an NLRP3 inhibitor with favorable pharmacokinetic properties. In a COVID-19 mouse model, it exhibited significant inhibitory effects. The assessment of its role in restraining IL-1β release and the concurrent activation of NLRP3 inflammatory proteins in THP-1 cells revealed its dose-dependent efficacy across multiple experiments. Notably, compound 7 exhibits high selectivity toward tumor necrosis factor-α (TNF-α). Even at elevated concentrations, it does not impact the production of TNF-α, signifying its specific activity against NLRP3 inflammasomes. Moreover, compound 7 effectively blocked the oligomerization of NLRP3 during ASC activation [59].

#### 4.1.2. Chloroquine and Hydroxychloroquine

As a drug exhibiting immunomodulatory properties, hydroxychloroquine has been utilized in the treatment of various autoimmune disorders. Chloroquine, known for its antagonistic activity against COVID-19, demonstrates effects on mouse bone marrow-derived macrophages. It diminishes NF-κB and MAPK activity, resulting in the reduced expression of IL-1β, IL-18, and NLRP3, thereby impeding the activation signal of NLRP3. Furthermore, chloroquine inhibits caspase-1 activation and the assembly of the ASC complex, thereby impeding the formation of the inflammatory complex and subsequently suppressing the assembly of NLRP3 inflammasomes [60]. Moreover, hydroxychloroquine appears to exhibit a role in counteracting the cytokine storm induced by COVID-19, primarily through its capability to reduce cytokine release [61].

#### 4.1.3. Dexamethasone

Dexamethasone exhibits multifaceted anti-inflammatory effects, not only by impeding vasodilation and immune cell migration but also through binding to glucocorticoid receptors in the cytoplasm, initiating an immune cell response, and reducing the transcription of pro-inflammatory cytokines like IL-1, IL-2, IL-6, IL-8, tumor necrosis factor, and interferon-γ. Additionally, it curtails pulmonary inflammation by inhibiting NLRP3 inflammasomes in the lungs [62]. Widely utilized in managing COVID-19, dexamethasone treatment has shown efficacy in human peripheral blood monocytes stimulated by the SARS-CoV-2S1 protein, leading to the mitigation of IL-1β imbalances and slight regulation of NLRP3 protein levels. Recent studies highlight the significant strides made by the steroid dexamethasone in reducing mortality among severe COVID-19 patients [63].

#### 4.1.4. DFV890

The novel compound DFV890 exhibits direct binding to NLRP3, effectively stabilizing it in an inactive state, thereby impeding NLRP3 activity and subsequently inhibiting the assembly of NLRP3 inflammasomes [64]. NLRP3 inflammasomes, known as intracellular polymer structures, play a pivotal role in triggering inflammatory responses, including the secretion of pro-inflammatory cytokines like IL-1β and IL-18, and eventual eosinophil death. The action of DFV890 disrupts this process, diminishing the release of inflammatory factors and mitigating cell death. Clinical trials involving DFV890 have showcased accelerated virus clearance, and its efficacy appears promising, potentially surpassing that of standard treatment (SOC), with marginally reduced mortality rates. Initiating DFV890 treatment early in hospitalized COVID-19 patients may contribute to improved treatment outcomes and reduced complications. These findings underscore the potential application of DFV890 in COVID-19 treatment, particularly in attenuating inflammatory reactions and enhancing therapeutic efficacy [65].

#### 4.1.5. MCC950

MCC950, also known as CRID3 or CP-456773, is a pharmacological agent specifically engineered to modulate NLRP3 (Nucleotide-binding Oligomerization Domain-like Receptor 3), a critical regulator in the immune response. Extensive research has demonstrated the pronounced efficacy of MCC950 in inhibiting the activation of the NLRP3 inflammasome, particularly when triggered by the SARS-CoV-2 N protein [22]. Additionally, it demonstrates efficacy in mitigating the inflammatory response induced by mouse cytomegalovirus (MCMV) infection and the miR-1929-3p inhibitor [66]. Moreover, MCC950 exhibits the capacity to reduce the expression of NLRP3 and caspase-1 proteins, alongside diminishing the production and secretion of IL-18 protein. In addition, MCC950 administration could efficiently diminish the expression of caspase-1 NLRP3 and reverse the elevated levels of IL-1β in human PBMCs exposed to the SARS-CoV-2 S1 protein [67]. The stimulation of SARS-CoV-2S1 led to the upregulation of tumor necrosis factor α, IL-6, IL-1β, and IL-8 production. However, pretreatment with CRID3 resulted in a notable reduction in IL-1β levels. In the presence of CRID3 (1 μM), the otherwise observed elevation in NLRP3 protein expression induced by S1 was significantly attenuated. Notably, a decrease in caspase-1 activity was observed upon pre-administration of CRID3 (1 μM) prior to S1 stimulation. These findings suggest the potential of MCC950 in mitigating the excessive inflammatory response induced by SARS-CoV-2 infection, potentially aiding in diminishing disease severity.

### 4.2. Immunomodulator Drugs

#### 25-HC@DDAB

Nanotechnology-based formulations like 25 hydroxycholesterol and 22 alkyldimethylammonium bromide (25-HC@DDAB) nanocapsules exhibit potential in treating COVID-19 by targeting NLRP3. These nanocapsules display selective accumulation in lung tissues and demonstrate efficacy in down-regulating NF-κB and SREBP2 signal pathways within peripheral blood mononuclear cells (PBMCs) derived from COVID-19 patients. This down-regulation results in reduced levels of inflammatory cytokines, the suppression of NLRP3 gene expression, and the inhibition of IL-1β secretion. Moreover, 25-HC@DDAB showcases inhibitory effects on cytokine release syndrome (CRS), which is observed in the peripheral blood mononuclear cells of individuals affected by SARS-CoV-2 infection [68].

### 4.3. Antidiabetic Drugs

#### Metformin

Metformin, a primary medication for type 2 diabetes management, activates phosphorylated AMPK, subsequently reducing the expression of pro-inflammatory cytokines like TNF-α, IL-6, and IL-1β. This action leads to the decreased activation of NLRP3 inflammasomes, thereby inhibiting lipopolysaccharide (LPS) and SARS-CoV-2-induced acute respiratory distress syndrome (ARDS). The primary mechanism underlying the prevention of NLRP3 inflammasome activation involves metformin’s ability to inhibit mitochondrial DNA synthesis induced by Toll-like receptors (TLRs). This inhibition blocks NLRP3 inflammasome activation both in vivo and in vitro, safeguarding mice from endotoxin-induced ARDS. Metformin’s primary molecular target is ETCCI, leading to reduced ATP production. Notably, it induces a more substantial decrease in ATP concentration in endotoxin-stimulated macrophages compared to unstimulated ones [69]. Additionally, the gene ablation of the ETCCI subunit Ndufs4 can similarly inhibit NLRP3 inflammasome activation and mitochondrial DNA synthesis induced by endotoxins [70]. Overall, these mechanisms collectively contribute to the reduction of acute respiratory distress syndrome induced by both lipopolysaccharide and SARS-CoV-2.

### 4.4. Lipid-Lowering Drugs

#### Statins

Statins, primarily prescribed for lowering cholesterol and preventing cardiovascular conditions, have gained attention for their potential immunomodulatory effects, notably in inhibiting NLRP3 inflammasomes. Following the onset of the COVID-19 pandemic, investigations have delved into the potential of statins to address the immune response triggered by the novel coronavirus. Recent findings suggest that statins might mitigate the cytokine storm observed in severe COVID-19 cases by engaging diverse molecular mechanisms, including the modulation of NF-κB signaling pathways and the suppression of NLRP3 inflammasomes [71].

### 4.5. Natural Products

#### 4.5.1. Colchicine

Colchicine, an accessible and cost-effective medication, possesses anti-inflammatory properties targeting NLRP3 inflammation. However, its efficacy regarding hospital stay, 28-day mortality, oxygenation support demand, or mortality has been found to be ineffective. It is worth noting that colchicine might diminish the formation of NLRP3 inflammasomes by inhibiting P2X7 receptors, exhibiting a modest inclination towards reducing hospitalization or mortality among COVID-19 patients [72].

#### 4.5.2. Curcumin

Curcumin, also called diferuloylmethane, is a principal curcuminoid of turmeric and has been demonstrated to inhibit the NLRP3 inflammasome observed in COVID-19 patients without any adverse effects [73]. Curcumin activates nuclear factor erythroid 2-related factor 2 (NRF2). The Nrf2 is a transcription factor that increases the expression of a number of antioxidant proteins under stress [74]. This transcription factor is highly expressed in the lungs, heart, liver, brain, and kidneys. One of the functions of Nrf2 is to inhibit the activity of NLRP3 inflammasomes as a basic signaling complex in exacerbating inflammation [75].

Recently, natural products have garnered increased attention for their potential in managing COVID-19. Certain constituents among these compounds demonstrate the ability to prevent the activation of NLRP3 inflammasomes. Compounds such as dihydroquercetin [76], resveratrol [77], quercetin [78], isoliquiritigenin [79], icariin [80], and oridonin [81] exhibit properties that suggest their suitability as promising candidates for regulating the activation of SARS-CoV-2-induced NLRP3 inflammasomes.

**Figure 3:**
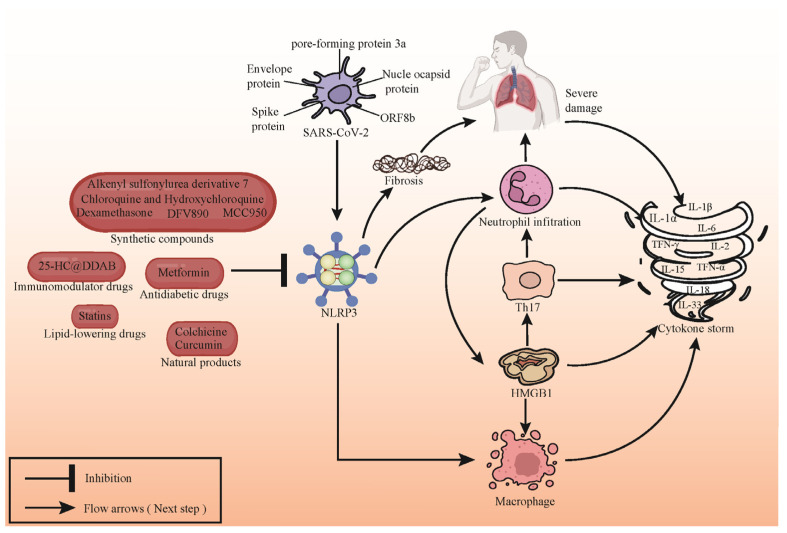
Compound targeting NLRP3 inhibits cytokine storm induced by SARS-CoV-2 infection. SARS-CoV-2 infection triggers activation of NLRP3 inflammasomes, leading to the accumulation and activation of immune cells. This provokes rapid migration of immune cells to infected sites, triggering inflammatory responses in lungs and other tissues, exacerbating the disease, and releasing inflammatory mediators such as IL-1β, IL-18, TNF-α, and IFN-γ, culminating in cytokine storms. Compounds targeting NLRP3 can mitigate SARS-CoV-2-induced inflammation by inhibiting the activation of NLRP3 inflammasomes. Representative NLRP3 inhibitors are depicted in the figure.

NLRP3 inhibitors demonstrate potential in modulating immune responses and serve as a pivotal intervention in managing cytokine storms triggered by novel coronavirus infection (Figure 3). By obstructing the NLRP3 pathway, these inhibitors exhibit efficacy in diminishing the inflammatory cascade and effectively regulating the hyperactivation of the immune system, consequently mitigating the extent of inflammation and associated pathophysiological manifestations. This therapeutic approach presents a promising avenue for attenuating immune-mediated damage induced by novel coronavirus infection. However, comprehensive research and rigorous clinical validation are imperative to ascertain its safety profile and therapeutic efficacy in clinical settings.

## 5. Discussion

As a key molecular mechanism in immune regulation, the NLRP3 inflammasome plays an important role in disease development and virus infection. In the context of viral infection, inflammasomes are implicated in a dual functionality, prompting a thorough investigation into their intricate role within the realm of antiviral immunity. On the one hand, the activation of inflammatory bodies is closely related to the response of immune cells to viral infection. Its activation can trigger the release of pro-inflammatory cytokines (such as IL-1 β and IL-18), thus promoting the activation of immune cells and virus clearance. This process may help to limit the replication and spread of the virus and play an important role in the early stages of infection. On the other hand, some studies have also pointed out that the excessive activation of inflammatory bodies may lead to an uncontrolled immune response, aggravate tissue damage and inflammatory responses, thus promoting the survival and spread of the virus. At this time, the inhibition of inflammatory bodies may help to reduce the inflammatory response and protect the host from excessive immune damage. In addition, the activity of inflammatory bodies may vary depending on the type of virus, the degree of infection, and the host’s immune status. This complexity makes the understanding of the role of inflammatory bodies in different viral infections more complex and diverse. Therefore, as an important immunomodulatory factor in the process of virus infection, the dual role of inflammatory bodies is worthy of further study.

In the pathophysiological landscape of SARS-CoV-2 infection, the mechanism of NLRP3 inflammasome has emerged as a focal point. NLRP3 assumes a critical role in immune modulation, rendering it a significant area of exploration within the realm of viral infections. Through the activation of pro-inflammatory cytokines IL-1 β and IL-18, NLRP3 exacerbates the inflammatory response, particularly within lung tissues, contributing to the profound pathological alterations following novel coronavirus infection. Presently, NLRP3 inhibitors are deemed a potential strategy for novel coronavirus infection treatment. Investigations into these inhibitors have revealed their capacity to modulate the NLRP3 pathway, effectively attenuating the exaggerated inflammatory response prompted by viral infection, thereby curtailing tissue damage, and fostering improved patient prognoses. Nonetheless, rigorous evaluation and comprehensive studies are imperative to ascertain the safety and efficacy profiles of these inhibitors for clinical applications. It is noteworthy that while the involvement of the NLRP3 pathway in viral infections has been preliminarily substantiated, its intricate regulatory mechanisms necessitate further exploration. Variances in NLRP3 pathway regulation across diverse individuals, disease stages, and therapeutic interventions underscore the need for comprehensive investigations to comprehensively comprehend its multifaceted role in divergent contexts. Moreover, the development of inhibitors confronts challenges, including dose determination, drug specificity, and the long-term safety aspects of their usage. Additionally, the inhibition of the NLRP3 pathway might elicit broader effects on immune responses, mandating a delicate balance between therapeutic efficacy and potential side effects in both research and clinical practice.

The investigation into the pathological mechanism involving the NLRP3 inflammasome in SARS-CoV-2 infection sheds light on the crucial role of the immune system in combating viral invasion. Our research delved into the potential mechanism of NLRP3 in SARS-CoV-2 infection, highlighting the likelihood that viral intrusion triggers the activation of the NLRP3 inflammasome, fostering the release of inflammatory factors that exacerbate the immune response and inflammatory cascades. This comprehension not only contributes to understanding COVID-19 pathogenesis but also offers essential leads for potential immunotherapeutic targets following novel coronavirus infection. Despite the widespread attention given to NLRP3 as a potential target for COVID-19 treatment, inhibitors targeting this pathway are still in early-stage research. Compounds like MCC950, β-hydroxybutyric acid, and metformin exhibit promising inhibitory effects on NLRP3, yet their safety and efficacy in clinical applications necessitate further validation. Additionally, drugs historically used for other conditions, such as chloroquine and hydroxychloroquine, are being investigated for their ability to inhibit NLRP3 activity, but their specific impacts on COVID-19 treatment warrant further evaluation. In summary, exploring the role of the NLRP3 inflammasome in novel coronavirus infection offers a crucial perspective for a deeper comprehension of immune response and inflammatory regulation. Future investigations should aim to explore the clinical viability, efficacy, and mechanisms of NLRP3 inhibitors in curbing the inflammatory process of COVID-19. These endeavors hold the potential to usher in a new era of treatment strategies for SARS-CoV-2 infections, offering more effective therapies for patients worldwide.

## Figures and Tables

**Table 1:** A list of antivirals developed to date against the NLRP3 inflammasome.

Drugs	Drug Category	The Role and Mechanism in the Treatment of COVID-19	References
Alkenyl sulfonylurea derivative 7	Synthetic compounds	It blocked the oligomerization of ASC during NLRP3 activation, and inhibited IL-1 β and IL-18 in a dose-dependent manner.	[59]
Chloroquine/ Hydroxychloroquine	Synthetic compounds	Chloroquine decreased the activity of NF-κB and MAPK, and inhibited the activation of caspase-1 and the formation of ASC complex.	[60,61]
Dexamethasone	Synthetic compounds	Reduce the release of pro-inflammatory factors and slightly regulate the protein level of NLRP3.	[62,63]
DFV890	Synthetic compounds	Inhibit the activity of NLRP3 by directly binding to NLRP3 and locking the protein in an inactive conformation.	[64,65]
MCC950	Synthetic compounds	Inhibit the activation of NLRP3 pathway and reduce the release of pro-inflammatory cytokines.	[22,66,67]
25-HC@DDAB	Immunomodulator drugs	Down-regulate NF-κB and SREBP2 signal pathways in PBMC derived from COVID-19 patients and reduce the expression of NLRP3 gene.	[68]
Metformin	Antidiabetic drugs	Inhibition of TLR-induced mitochondrial DNA synthesis, thereby blocking the activation of NLRP3 inflammatory bodies in vivo and in vitro.	[69,70]
Statins	Lipid-lowering drugs	Blocking NF-κB and NLRP3 inflammatory bodies.	[71]
Colchicine	Natural products	Inhibition of P2X7 receptor reduces the formation of inflammatory bodies in NLRP3.	[72]
Curcumin	Natural products	Inhibit the activity of NLRP3.	[73,74,75]

## Data Availability

No new data were created or analyzed in this study. Data sharing is not applicable to this article.
